# Successfully Reducing Sitting Time Can Improve Metabolic Flexibility

**DOI:** 10.1111/sms.70113

**Published:** 2025-08-09

**Authors:** Taru Garthwaite, Tanja Sjöros, Saara Laine, Mikko Koivumäki, Henri Vähä‐Ypyä, Jooa Norha, Petri Kallio, Maria Saarenhovi, Eliisa Löyttyniemi, Harri Sievänen, Noora Houttu, Kirsi Laitinen, Kari K. Kalliokoski, Tommi Vasankari, Juhani Knuuti, Ilkka Heinonen

**Affiliations:** ^1^ Turku PET Centre University of Turku, Turku University Hospital, and Åbo Akademi University Turku Finland; ^2^ The UKK Institute for Health Promotion Research Tampere Finland; ^3^ Department of Clinical Physiology and Nuclear Medicine University of Turku and Turku University Hospital Turku Finland; ^4^ Paavo Nurmi Centre and Unit for Health and Physical Activity University of Turku Turku Finland; ^5^ Department of Biostatistics University of Turku and Turku University Hospital Turku Finland; ^6^ Institute of Biomedicine and Food and Nutrition Research Center University of Turku Turku Finland; ^7^ Faculty of Medicine and Health Technology Tampere University Tampere Finland

**Keywords:** energy metabolism, metabolic flexibility, metabolic syndrome, physical activity, sedentary behavior

## Abstract

Impaired metabolic flexibility (MetFlex; the ability to regulate substrate oxidation) and sedentary behavior are both linked to cardiometabolic diseases, but the relationship between the two is not fully elucidated. This study investigated the effects of reduced sedentary time on MetFlex. Sixty‐four sedentary adults with metabolic syndrome were randomized into intervention (INT, *n* = 33) and control (CON, *n* = 31) groups. INT aimed to reduce sedentary time by 1 h/day by increasing standing and non‐exercise physical activity (PA) for 6 months, with continuous accelerometry. Substrate oxidation and MetFlex [ΔRER (respiratory exchange ratio)] from fasting to hyperinsulinemic‐euglycemic clamp and low‐ to maximal‐intensity exercise were assessed with indirect calorimetry. Intervention effects between INT and CON were analyzed with mixed models, and secondary analyses examined the effects based on accelerometer‐derived behavior changes. INT reduced sedentary time by 41 min/day. Neither insulin‐ nor exercise‐stimulated MetFlex changed in INT or CON, but carbohydrate oxidation during maximal exercise changed differently, favoring INT [INT +2.6 (95% CI: −6.1, 0.8), CON −1.4 (−2.1, 4.9) mg/kg/min; group * time *p* = 0.03]. In secondary analyses, those who successfully reduced sedentary time by at least 30 min/day (*n* = 34) improved insulin‐stimulated MetFlex and low‐intensity exercise fat oxidation compared to the continuously sedentary (*n* = 30) [ΔRER +0.03 (−0.01, 0.07) vs. −0.02 (−0.06, 0.03); and FATox +0.2 (−0.3, 0.7) vs. −0.4 (−1.0, 1.0) mg/kg/min, group * time *p* < 0.05]. Changes in insulin‐stimulated MetFlex correlated with changes in standing and insulin sensitivity. Thus, successfully reducing daily sitting by at least half an hour can improve MetFlex, with parallel insulin sensitivity enhancements, and aid in cardiometabolic disease prevention in risk populations.

**Trial Registration:** https://www.clinicaltrials.gov/study/NCT03101228

## 1 Introduction

Metabolic flexibility (MetFlex) represents the ability to match fuel oxidation to availability and metabolic demand [1]. Dysregulated lipid and carbohydrate (CHO) metabolism and blunted substrate switching in response to changes in metabolic and physiological conditions (=metabolic inflexibility) are features of obesity, insulin resistance, metabolic syndrome, and type 2 diabetes [2, 3, 4]. Although not one of the criteria of metabolic syndrome per se, metabolic inflexibility is a common characteristic alongside the established markers of metabolic syndrome (central obesity, dyslipidemia, elevated fasting glucose, elevated blood pressure) [5]. One of the representations of metabolic inflexibility in metabolic syndrome is impaired insulin‐mediated substrate switching, and, consequently, metabolic inflexibility is proposed as a major contributor to the development of insulin resistance [6]. Together, metabolic inflexibility and metabolic syndrome considerably increase the risk of long‐term health complications, including type 2 diabetes and cardiovascular disease [6]. Sedentary time and physical inactivity similarly associate with adverse cardiometabolic outcomes and an increased disease risk [7, 8]; however, the relationship between habitual physical activity (PA) behaviors and MetFlex is unclear.

Previous studies from us and others propose sedentary time and PA as determinants of MetFlex, but current evidence relies mostly on observational and short‐term experimental studies, and structured exercise interventions [9, 10, 11, 12]. Evidence on the causality between free‐living activity behaviors and energy metabolism regulation is yet to be established in long‐term intervention studies, particularly in populations with an increased cardiometabolic risk. Studying the causality between sedentary time and metabolic inflexibility in the intervention setting will advance the understanding of the role of lifestyle factors in energy metabolism and the progression of metabolic diseases.

The primary aim was to investigate the effects of a 6‐month sedentary time reduction intervention on MetFlex in sedentary adults with metabolic syndrome. A secondary aim was to examine the effects based on the measured change in sedentary time. Reducing sedentary time, without adding intentional exercise, was hypothesized to improve MetFlex.

## 2 Materials and Methods

### 2.1 Study Design

This free‐living parallel‐group randomized controlled trial consisted of a 1‐month screening phase and a 6‐month intervention phase. Accelerometers were used continuously throughout both phases, and outcome measurements were performed at baseline after screening and repeated after the intervention. The protocol was approved by the Ethics Committee of the Hospital District of Southwest Finland (16/1801/2017), and the data were collected at the Turku PET Centre (Turku, Finland) between 2017 and 2020. The study conformed to the standards set by the Declaration of Helsinki (Version 2013) and is registered at https://www.clinicaltrials.gov/study/NCT03101228. Informed consent was obtained from all participants in writing.

### 2.2 Participants

Participants were recruited with newspaper advertisements and bulletin leaflets. As previously reported [13], the inclusion criteria were age 40–65 years; physical inactivity [< 120 min/week of self‐reported moderate‐to‐vigorous PA (MVPA)]; sedentary time ≥ 10 h/day or 60% of accelerometer wear time/day during screening; BMI 25–40 kg/m^2^; and the fulfillment of metabolic syndrome criteria (at least three of the following: central obesity; elevated fasting glucose, triglycerides, systolic or diastolic blood pressure; and/or low HDL‐cholesterol) [5]. The exclusion criteria were diagnosed diabetes, history of any cardiac disease, abundant alcohol consumption, cigarette smoking, use of narcotics or snuff tobacco, depressive or bipolar disease, and any chronic disease or condition that could endanger participant safety or study procedures or interfere with the interpretation of results. Sixty‐four participants were recruited according to the sample size calculation for the primary outcome of the entire research project (whole‐body insulin sensitivity) [13]. The results presented here are for secondary outcomes.

### 2.3 Intervention

After the screening and baseline measurements, participants were allocated into intervention (INT; *n* = 33) and control (CON; *n* = 31) groups based on a randomization code generated by a statistician with SAS 9.4 (SAS Institute Inc., Cary, NC, USA). Random permuted blocks were used in a 1:1 ratio, separately for men and women.

The intervention has been described in detail previously [13]. In summary, INT aimed to reduce sedentary time by 1 h/day compared to the individually determined baseline by increasing standing and non‐exercise PA. The preferred ways were individually discussed with participants in 1‐h counseling sessions to find feasible ways to incorporate into the participants' daily lives, and could include, for example, using standing desks, choosing stairs over elevators, and light walking. The specific strategies to reduce sedentary time were discussed more in‐depth with each participant individually in the counseling sessions. Participants were encouraged to consider their existing habits already involving PA, as well as potential new, enjoyable, and/or easy ways to reduce sedentary time and incorporate more activity into daily routines. Things that might support or prevent achieving the goals were also identified, together with potential solutions to challenges. The participants were contacted by phone 2–3 times during the intervention, and they visited the research center at 3 months to receive support with the goals and to ensure that the accelerometers and the mobile application were working properly. CON was guided to maintain usual habits during the intervention, and they were provided an opportunity to receive guidance for behavior change in personal counseling sessions similar to INT after the study conclusion. Both groups used accelerometers (Movesense, Suunto, Vantaa, Finland) throughout the 6‐month intervention. The accelerometers were connected to a mobile application (ExSed, UKK Terveyspalvelut Oy, Tampere, Finland) to enable self‐monitoring of the individual goals for daily sedentary time and PA. For INT, 1 h was subtracted from baseline sedentary time and added to standing and PA according to participants' preferences (with a maximum of 20 min added to MVPA), and for CON, the goals were equal to the baseline values.

### 2.4 Accelerometry

Triaxial hip‐worn accelerometers were used during waking hours throughout the 4‐week screening (UKK AM30, UKK Terveyspalvelut, Tampere, Finland) and the 6‐month intervention (Movesense, Suunto, Vantaa, Finland) phases. Wear time 10–19 h/day was considered valid. Two different accelerometers were used because UKK AM30 allowed the blinding of participants to their activity data during screening, with the aim of capturing their normal behavior to ensure eligibility to enter the intervention study. Conversely, Movesense was connected to an application to enable self‐monitoring and fulfillment of individually set goals during the intervention. UKK AM30 recorded raw acceleration data at a sampling frequency of 100 Hz, and the collected raw data was stored on a hard disk for analysis. Due to a longer collection period and thus a larger amount of data, Movesense recorded raw data at 52 Hz. For the data to be transferred to a cloud server for analysis, the participants were required to regularly access the connected app. The raw accelerometer data from both accelerometers was analyzed in 6‐s epochs with validated mean amplitude deviation (MAD) [14] and angle for posture estimation (APE) methods, which can identify postures with 90% accuracy in free‐living conditions [15]. Sedentary time was defined as < 1.5 metabolic equivalents (METs) and APE ≥ 11.6°, standing as < 1.5 METs and APE < 11.6°, light‐intensity PA (LPA) as 1.5–< 3.0 METs, and MVPA as ≥ 3.0 METs.

### 2.5 Cardiorespiratory Fitness

Cardiorespiratory fitness was assessed with a graded maximal cycle ergometry test (eBike EL Ergometer + CASE v6.7, GE Medical Systems Information Technologies Inc., Milwaukee, WI, USA) according to the previously described protocol [16]. In short, the load started at 25 W and was increased by 25 W every 3 min until exhaustion. VO_2max_ (mL/kg/min), VO_2max_ per fat‐free mass (FFM) (mL/kg_FFM_/min), and maximal power output (W) were determined as outcomes.

### 2.6 Hyperinsulinemic‐Euglycemic Clamp (HEC)

HEC was performed after an overnight fast as previously described [13]. Insulin (Actrapid, 100 U/mL, Novo Nordisk, Bagsvaerd, Denmark) was administered at a steady 40 mU/min/m^2^ body surface area rate after priming with higher doses. A 20% glucose infusion was started 4 min after starting the insulin infusion. The rate was adjusted according to blood sampling every 5–10 min to maintain ~5.0 mmol/L glucose concentration. Whole‐body glucose uptake (mg/kg/min) was calculated in 20‐min intervals from steady‐state glucose values as a measure of whole‐body insulin sensitivity.

### 2.7 Indirect Calorimetry

As described in detail previously [9], respiratory gas exchange was measured with a ventilated hood system (Quark RMR + OMNIA, COSMED, Rome, Italy) for 20 (SD 1) min in an overnight‐fasted state, and for 15 (SD 2) min during HEC, starting 29 (SD 8) min after HEC start. MetFlex was defined as the change in respiratory exchange ratio (RER = VCO_2_/VO_2_) from fasting to insulin stimulation (ΔRER). Respiratory gases were also collected breath by breath during the cardiorespiratory fitness test with a mask (Vyntus CPX, CareFusion, Yorba Linda, CA, USA). Exercise‐stimulated MetFlex was calculated as ΔRER from low‐ to maximal‐intensity exercise. Carbohydrate (CHOox) and fat oxidation (FATox) were calculated from respiratory gases in fasting and insulin‐stimulated states [17], and during exercise [18], assuming negligible protein oxidation.

### 2.8 Diet

Dietary intake was assessed with 4‐day food diaries (including 1 weekend day). Food quotient (FQ; theoretical expected RER if dietary macronutrients were completely oxidized) was calculated from macronutrient intake [19]. RER:FQ ratio was calculated to represent substrate oxidation relative to macronutrient intake; e.g., a value > 1 suggests lower fat oxidation relative to intake [20].

### 2.9 Metabolic and Anthropometric Outcomes

Plasma insulin, glucose, triglycerides, free fatty acids (FFA), and lactate were determined from venous blood samples after ≥ 10 h of fasting. Blood samples were also collected from all participants during HEC (~80 min from the start) to determine insulin‐stimulated FFA suppression and lactate increase. Additional blood samples from timepoints ~115, ~135, and ~155 min were available for a subsample of 44 participants [21], which were used to calculate the area under the curve (AUC) for FFA and lactate with the trapezoidal rule.

Body weight, fat mass, FFM, and body fat‐% were assessed with air displacement plethysmography (Bod Pod, COSMED USA Inc., Concord, CA, USA) after fasting ≥ 4 h. Height, BMI, and waist circumference were measured with standard methods.

### 2.10 Statistics

Means (SD), or medians (lower quartile Q1, upper quartile Q3) for non‐normally distributed outcomes [whole‐body glucose uptake; fasting insulin, FFA, lactate, energy expenditure (EE), and RER; insulin‐stimulated FFA, lactate, and MetFlex; and CHOox at low exercise intensity] were calculated. Intervention effects and within‐ and between‐group changes over time in INT and CON were examined in the primary analyses with sex‐adjusted linear mixed models for repeated measurements (including group, time, and group * time‐interaction), using the Tukey–Kramer method for multiple comparisons. Results are reported as model‐based means with 95% confidence intervals (CI). Correlations between changes (Δ) during the intervention among all participants were analyzed with Spearman's rank correlation. Statistical significance was set at *p* < 0.05 (two‐tailed). SAS 9.4, IBM SPSS Statistics 27.0 (IBM Corp., Armonk, NY, USA), GraphPad Prism 5.01 (GraphPad Software, San Diego, CA, USA), and JMP Pro 16.0.0 (SAS Institute Inc., Cary, NC, USA) were used for analyses and figure creation.

For secondary analyses, participants were re‐divided into two groups according to the measured sedentary time change (regardless of the original allocation): ‘reducers’ with a ≥ 30‐min/day reduction in sedentary time (*n* = 34), and ‘continuously sedentary’ with a smaller reduction or an increase in sedentary time (*n* = 30) [13]. The cut‐point was chosen to form relatively equally sized groups. Participants with missing accelerometer data (*n* = 8) were allocated according to the original randomization. The linear mixed model analyses were repeated with these groups.

## 3 Results

Table 1 presents the baseline characteristics. One participant in INT and three in CON discontinued the study (CONSORT Flow Diagram in Figure S1).

**TABLE 1:** Baseline characteristics of participants.

	Intervention	Control
*n* (%)	33 (52)	31 (48)
Sex, women, *n* (%)	20 (69)	17 (55)
Age, years	59.3 (6.0)	57.2 (7.5)
Weight, kg	92.4 (16.6)	94.1 (15.8)
BMI, kg/m^2^	31.5 (4.0)	31.7 (4.6)
Waist circumference, cm	111.1 (11.6)	110.7 (11.1)
Body fat‐%	43.1 (8.0)	43.1 (8.0)
Fat mass, kg	39.8 (10.4)	40.9 (11.1)
Fat‐free mass, kg	52.6 (11.9)	53.2 (9.8)
Activity and fitness outcomes		
Accelerometer wear time, h/day	14.47 (0.96)	14.60 (1.00)
Sedentary time, h/day	10.02 (0.92)	10.06 (1.11)
Standing, h/day	1.81 (0.61)	1.76 (0.57)
LPA, h/day	1.67 (0.40)	1.81 (0.48)
MVPA, h/day	0.96 (0.31)	0.97 (0.34)
Total PA, h/day	2.64 (0.52)	2.78 (0.72)
Steps/day	5204 (1910)	5091 (1760)
Breaks in sedentary time/day	28 (8)	29 (8)
VO_2__max_, mL/kg/min^a^	22.7 (5.0)	22.8 (4.3)
VO_2__max_, mL/kg_FFM_/min^a^	40.0 (5.9)	39.9 (6.4)
Dietary intake		
Energy intake, kcal/day	1737 (383)	1861 (412)
CHO, g/day	165.0 (46.6)	180.9 (50.6)
Fat, g/day	75.3 (22.8)	83.5 (22.0)
SFA, g/day	26.7 (8.8)	30.4 (8.7)
MUFA, g/day	26.5 (9.0)	28.4 (9.6)
PUFA, g/day	12.1 (5.0)	13.0 (5.1)
Protein, g/day	77.4 (20.7)	79.5 (20.0)
CHO, % of energy intake/day	38.8 (8.7)	39.5 (6.5)
Fat, % of energy intake/day	38.2 (7.6)	39.5 (5.1)
Protein, % of energy intake/day	18.1 (2.8)	17.5 (2.9)
FQ	0.83 (0.03)	0.83 (0.02)
Metabolic outcomes		
Insulin, pmol/L		
Fasting^b^	62.5 (48.6, 93.8)	76.4 (46.9, 123.3)
HEC	495.9 (93.9) ***	512.1 (108.9) ***
Glucose, mmol/L		
Fasting	5.9 (0.5)	5.8 (0.4)
HEC	5.1 (0.4) ***	5.3 (0.6) ***
Whole‐body glucose uptake, mg/kg/min	2.7 (1.9, 3.8)	2.4 (1.7, 3.8)
Free fatty acids, mmol/L		
Fasting	0.58 (0.50, 0.76)	0.56 (0.44, 0.66)
HEC	0.12 (0.07, 0.17) ***	0.11 (0.05, 0.22) ***
AUC^c^	36.7 (11.9)	36.4 (13.8)
Lactate, mmol/L		
Fasting	1.0 (0.9, 1.6)	1.0 (0.8, 1.2)
HEC	1.2 (1.1, 1.5) **	1.2 (1.0, 1.4) ***
AUC^c^	189.5 (44.8)	174.4 (39.7)
EE, kcal/day		
Fasting^d^	1585 (1141, 1861)	1681 (1489, 1897)
HEC	1806 (336) ***	1769 (262) ***
CHO oxidation, mg/kg/min		
Fasting^d^	2.8 (1.0)	2.2 (0.7)
HEC	2.8 (1.2)	2.3 (0.9)
Fat oxidation, mg/kg/min		
Fasting^d^	0.3 (0.4)	0.5 (0.4)
HEC	0.3 (0.5)	0.5 (0.4)
RER		
Fasting^d^	0.94 (0.87, 0.99)	0.89 (0.84, 0.97)
HEC	0.94 (0.10)	0.90 (0.08)
ΔRER (HEC − fasting)^d^	−0.01 (−0.04, 0.02)	0.00 (−0.04, 0.05)
RER:FQ^d^	1.13 (0.11)	1.08 (0.10)
Low‐intensity exercise^b^		
Power output, W	25	25
EE, kcal/min	2.9 (0.8)	3.0 (0.5)
CHO oxidation, mg/kg/min	1.9 (1.2, 2.8)	2.2 (1.0, 3.1)
Fat oxidation, mg/kg/min	2.3 (0.9)	2.5 (1.0)
RER	0.73 (0.05)	0.74 (0.04)
Maximal exercise^a^		
Max power output, W	128 (33)	132 (30)
EE, kcal/min	10.6 (2.7)	11.0 (2.2)
CHO oxidation, mg/kg/min	39.5 (9.3)	38.9 (8.7)
Fat oxidation, mg/kg/min	−4.4 (2.3)	−4.1 (2.2)
RER	1.13 (0.06)	1.12 (0.06)
ΔRER (maximal − low‐intensity exercise)	0.40 (0.07)	0.37 (0.07)

*Note:* Values are means (SD), or medians with upper and lower quartiles (Q1, Q3) for non‐normally distributed outcomes. **p < 0.01, ***p < 0.001 between fasting and insulin stimulation.

Abbreviations: AUC, area under curve; CHO, carbohydrate; EE, energy expenditure; FFM, fat‐free mass; FQ, food quotient; HEC, hyperinsulinemic‐euglycemic clamp; LPA, light‐intensity physical activity; MUFA, monounsaturated fatty acids; MVPA, moderate‐to‐vigorous physical activity; PA, physical activity; PUFA, polyunsaturated fatty acids; RER, respiratory exchange ratio; SFA, saturated fatty acids; VO_2__max_, maximal oxygen consumption.

^a^ Data available for 58 participants.

^b^ Data available for 63 participants.

^c^ Data available for 44 participants.

^d^ Data available for 62 participants.

### 3.1 Sedentary Time and PA

The intervention was successful in reducing sedentary time, as reported earlier [11]. In summary, INT reduced sedentary time from baseline by 41 (95% CI: 17, 65) min/day on average, primarily through the combination of increased LPA and standing, as intended [21 (2, 41) min/day]. INT also increased MVPA by 20 (11, 28) min/day, while CON maintained baseline values of sedentary time and PA (group * time *p* < 0.05 for all). Both groups increased steps, but INT more so (INT +3300 vs. CON +1600 steps/day; group * time *p* = 0.001). There was large interindividual variation in changes in daily sedentary time during the intervention (Figure S2).

### 3.2 MetFlex and Substrate Metabolism

**FIGURE 1:**
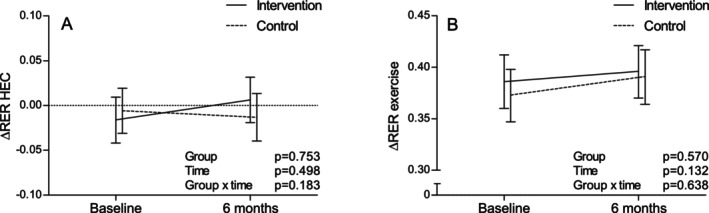
Changes during the intervention in (A) insulin‐ and (B) exercise‐stimulated metabolic flexibility (ΔRER) in the intervention (*n* = 33) and control (*n* = 31) groups (model‐based means with 95% confidence intervals). HEC, hyperinsulinemic‐euglycemic clamp; RER, respiratory exchange ratio.

Neither insulin‐ nor exercise‐stimulated MetFlex changed during the intervention in either group in the primary analyses; nor were there between‐group differences (Figure 1).

**FIGURE 2:**
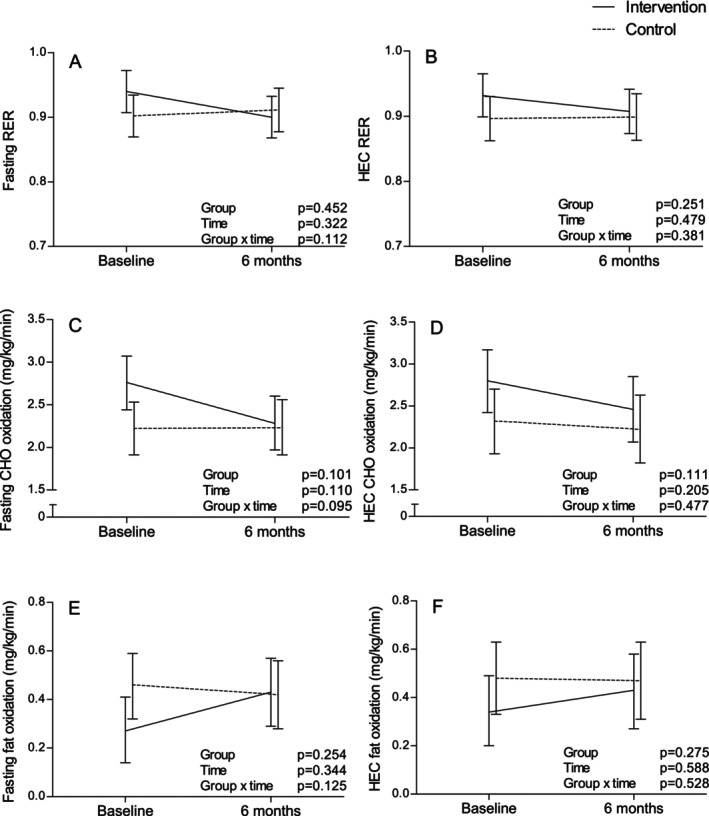
Changes during the intervention in (A) fasting RER, (B) insulin‐stimulated RER, (C) fasting carbohydrate oxidation, (D) insulin‐stimulated carbohydrate oxidation, (E) fasting fat oxidation, and (F) insulin‐stimulated fat oxidation in the intervention (*n* = 33) and control groups (*n* = 31) (model‐based means with 95% confidence intervals). CHO, carbohydrate; HEC, hyperinsulinemic‐euglycemic clamp; RER, respiratory exchange ratio.

Changes in substrate oxidation variables were not statistically significantly different between groups either; although, fasting CHOox tended to decrease and fasting FATox tended to increase in INT compared to CON (Figure 2C,E). Tables S1–S3 report the numerical estimates of Figures 1 and 2.

CHOox at maximal exercise intensity changed differently between groups as INT increased and CON decreased it by 2.6 (95% CI: −0.8, 6.1) and 1.4 (−4.9, 2.1) mg/kg/min, respectively (group * time *p* = 0.03). No other changes in RER and substrate oxidation during exercise were observed (Table S2). Changes in insulin‐ and exercise‐stimulated MetFlex did not correlate with each other (Table S4).

Fasting lactate decreased in both groups (time *p* = 0.008). The decrease was greater in INT, but the difference in change between groups was marginally non‐significant [INT −0.19 (95% CI: −0.33, −0.04) mmol/L, CON −0.03 (−0.18, 0.12) mmol/L; group * time *p* = 0.054]. Changes in fasting lactate correlated with changes in insulin‐stimulated MetFlex and fasting RER (Table S4). Decreased fasting lactate also correlated with increased fasting FATox and decreased fasting CHOox (*r* = −0.38, *p* = 0.004 and *r* = 0.45, *p* < 0.001; respectively). The intervention had no effect on fasting plasma glucose or FFA concentration. No changes or differences between groups were observed in FFA and lactate concentrations or AUC during HEC either (data not shown).

### 3.3 Secondary Analyses

**FIGURE 3:**
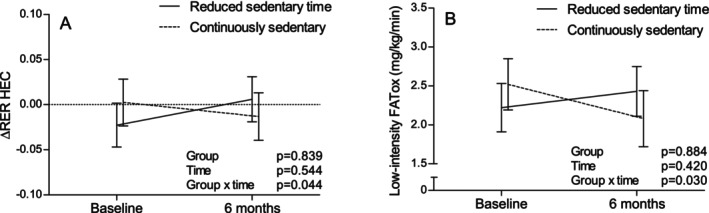
Changes during the intervention in (A) insulin‐stimulated metabolic flexibility (ΔRER) and (B) fat oxidation during low‐intensity exercise in those who successfully reduced sedentary time (≥≈30 min/day reduction in sedentary time, *n* = 34) and the continuously sedentary group (*n* = 30) (model‐based means with 95% confidence intervals). HEC, hyperinsulinemic‐euglycemic clamp; RER, respiratory exchange ratio.

The change in insulin‐stimulated MetFlex and FATox at low‐intensity exercise was different between groups in favor of the ‘reducers’ compared to the ‘continuously sedentary’ (group * time *p* = 0.04 and *p* = 0.03, respectively; Figure 3).

A group * time‐effect (*p* = 0.04) was also observed in fasting RER, which was driven primarily by the ‘reducers’ higher baseline value. Adjustment for baseline fasting RER slightly mitigated the MetFlex interaction effect (group * time *p* = 0.05). Low‐intensity exercise RER decreased by −0.01 (95% CI: −0.03, 0.00) on average during the intervention, with no difference between the groups (time *p* = 0.04; Table S3).

### 3.4 Correlations Between Changes

#### 3.4.1 Sedentary Time and PA

**FIGURE 4:**
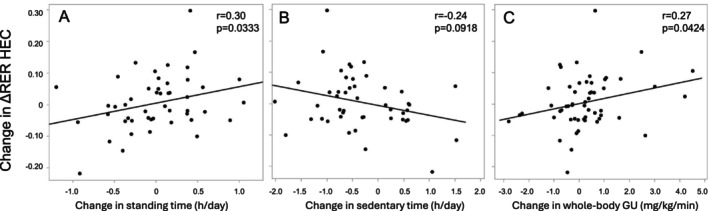
Spearman's correlations between changes during the intervention in insulin‐stimulated metabolic flexibility (ΔRER) and (A) standing time, (B) sedentary time, and (C) whole‐body glucose uptake among all participants (*n* = 64). GU, glucose uptake; HEC, hyperinsulinemic‐euglycemic clamp; RER, respiratory exchange ratio.

Improved insulin‐stimulated MetFlex correlated with increased standing, with a reverse trend observed for sedentary time (Figure 4A,B). Changes in neither exercise‐stimulated MetFlex (Table S4) nor substrate oxidation variables (data not shown) correlated with changes in activity outcomes.

#### 3.4.2 Insulin Sensitivity

Changes in whole‐body insulin sensitivity [13] correlated positively with changes in insulin‐stimulated MetFlex (Figure 4C) and RER (Table S4). Changes in insulin sensitivity also correlated with changes in CHOox and FATox during HEC (*r* = 0.42, *p* = 0.001, and *r* = −0.34, *p* = 0.01; respectively) and with changes in sedentary time, standing, LPA, steps, weight, and body composition (Table S4).

#### 3.4.3 Weight and Body Composition

Changes in weight and body composition [13] correlated with changes in exercise‐stimulated MetFlex and RER (Table S4) and with changes in substrate oxidation at maximal exercise intensity (data not shown). Changes in weight and adiposity also correlated with changes in sedentary time, LPA, and steps (Table S4).

#### 3.4.4 Diet

Changes in the intake of total fat, saturated (SFA) and polyunsaturated fatty acids (PUFA), and protein [22] correlated positively, and changes in RER:FQ ratio inversely, with changes in insulin‐stimulated MetFlex (*r* = 0.31, *r* = 0.29, *r* = 0.28, *r* = 0.31, and *r* = −0.32, respectively; *p* < 0.05 for all).

## 4 Discussion

This study shows that daily activity behaviors play a role in energy metabolism regulation. Although the intervention itself did not result in significant differences between INT and CON, the secondary analyses suggest that successfully reducing daily sedentary time by 30 min or more (on average about 60 min/day) can improve MetFlex in sedentary adults with an increased cardiometabolic risk, compared to a continuously sedentary lifestyle. As improved MetFlex correlated with increased standing and improved insulin sensitivity, reducing sedentary time and increasing even very light‐intensity activity may be beneficial for glucose and lipid metabolism, potentially alleviating a further deterioration of metabolic outcomes in risk populations. A larger reduction in sedentary time and/or increase in the amount and intensity of PA is likely needed for more pronounced improvements in overall metabolic health, however.

Sedentary time and PA are proposed to modulate MetFlex, but current evidence relies mostly on observational and short‐term experimental studies and exercise interventions [10, 12]. In support of activity behaviors as determinants of MetFlex, we previously showed that higher accelerometer‐assessed sedentary time associates with metabolic inflexibility and more PA, even of light intensity, with better MetFlex [9]. Similarly, changes in habitual PA level by training, detraining, or bed rest have been shown to modify MetFlex [11]. Long‐term free‐living intervention studies targeting reductions in sedentary time, without intentional exercise training, are needed to investigate the effects of habitual activity on MetFlex.

Several interventions have, however, reported improvements in MetFlex or substrate metabolism following exercise training, alone or together with diet modification. For example, 3–12 months of moderate‐intensity exercise is consistently reported to improve MetFlex in adults with obesity or type 2 diabetes [23, 24, 25]. In contrast, exercise training did not affect MetFlex in overweight or healthy men, despite improved insulin sensitivity [26, 27], indicating that existing metabolic impairments and health status may influence the capacity of exercise to improve MetFlex. Our study adds novel findings to the evidence from exercise studies, as the secondary analyses suggest that modifying daily activity level and increasing habitual PA, not exercise training per se, may also positively impact MetFlex in adults with an increased cardiometabolic risk.

Exercise interventions have often reported beneficial effects specifically on FATox [28, 29, 30]. Although our intervention itself was unable to affect lipid metabolism, successfully reducing daily sedentary time by at least half an hour may improve FATox during low‐intensity activity. We showed previously that more standing and PA are associated with higher FATox both in a fasting state and during low‐intensity exercise [9], and now observed a borderline significant correlation (*p* = 0.06) between increases in standing and low‐intensity exercise FATox. Previous low‐intensity exercise interventions have also improved FATox [28, 31], while reduced structured and spontaneous PA has resulted in lower FATox [32]. In contrast to moderate‐ to high‐intensity exercise, CHOox appears unaffected by low‐intensity exercise [28]. Interestingly, the intervention increased CHOox at maximal exercise compared to CON; however, the increase correlated with changes in weight, adiposity, and fitness, unlike changes in low‐intensity exercise FATox (data not shown). These findings suggest that reducing sedentary time and increasing even very light‐intensity activity may beneficially impact lipid metabolism. Altering daily sedentary and activity behaviors is possibly a more easily achievable way to enhance energy, particularly lipid, metabolism than exercise training for sedentary, inactive individuals. Furthermore, as the intervention did not improve cardiorespiratory fitness [16] or blood pressure [33], it seems that particularly metabolic health, over cardiovascular, could benefit from such low‐intensity interventions.

We reported previously that successfully reducing sedentary time improves insulin sensitivity [13, 21]. Now we show that improved insulin sensitivity also correlates with improvements in MetFlex, in line with other studies [23, 24, 29]. An important feature in the development of insulin resistance is mitochondrial dysfunction [34]. Blood lactate, which is considered an indirect marker of mitochondrial function and closely relates to substrate metabolism [3, 34], decreased in this study. Changes in lactate correlated with changes in MetFlex, FATox, and CHOox; thus indirectly suggesting improvements in mitochondrial function potentially underlying improved substrate metabolism and insulin sensitivity.

Improved insulin sensitivity also correlated with reduced sedentary time and increased standing, LPA, and steps, but interestingly only increased standing correlated with improved MetFlex. We showed previously that standing was associated with whole‐body and muscle insulin sensitivity cross‐sectionally [35, 36]. Body composition was a more important determinant of muscle insulin sensitivity than any (in)activity outcomes; however, standing was associated with whole‐body insulin sensitivity markers independent of adiposity [35]. Similarly, changes in sedentary time, LPA, and steps, but not standing, correlated with changes in weight and body composition in this study. Moreover, reduced weight and adiposity correlated with improved insulin sensitivity and exercise‐ but not insulin‐stimulated MetFlex. Altogether, these studies suggest that sedentary time and higher‐intensity PA may affect insulin sensitivity through effects on weight and body composition, but adiposity may not be the key modulator of the associations between standing, insulin sensitivity, and insulin‐stimulated MetFlex. Based on our intervention and cross‐sectional results, it could be speculated that this relationship between standing in particular (vs. movement and more intense PA) and metabolic benefits could be explained by the effects of standing on lipid metabolism, at least in part. Standing activates particularly hamstring muscles, which include primarily oxidative muscle fibers favoring fat metabolism [37, 38], and specifically low‐intensity muscle contractions increase skeletal muscle lipoprotein lipase activity [39]. Alterations in the delivery and uptake of fatty acids into mitochondria have indeed been proposed as a potential mechanism underlying the health benefits of low‐intensity activity [40], but pathways explaining standing‐induced benefits in particular are yet to be more thoroughly explored. Moreover, MetFlex responses to varying metabolic challenges likely have different underlying mechanisms, as also suggested by previous findings [41], since changes in insulin‐ and exercise‐stimulated MetFlex correlated differently with body composition changes, and there was no correlation between the two.

The unintended step increase in CON and the decreased intake of total and saturated fat in comparison to INT [22] could partly explain the lack of difference in MetFlex in the primary analyses. These behavior changes in the CON group as well may be due to a participation effect, since, even without receiving the intervention, the awareness of behaviors and health outcomes being monitored and measured may lead to behavior modifications [42]. The secondary analyses based on the actual, measured behavior changes therefore provide valuable and translatable insights into the effects of successfully modified daily activities, although not able to determine what specifically led to the modifications. It could be speculated that only the use of the mobile application, without any intervention, may have tempted and inspired CON to change activity behaviors as well. Even if not the intention of this study per se, it is positive from a public health perspective if only the use of an activity tracking application is enough to elicit changes in activity behaviors in inactive and sedentary populations.

The participants were instructed to maintain usual dietary habits during the study, but participating in a behavioral health intervention may have inspired them to pay attention to other health‐related behaviors outside of the primary aims as well. This may have led to the slight differences in fat intake between groups. Exercise and physical inactivity can alter the oxidation of specific types of dietary fats as well [43, 44], and the amount or differences in fatty acid type partitioning may impact MetFlex [45]. Although here it was not possible to distinguish between the oxidation of saturated and unsaturated dietary fat, changes in fat intake and in the ability to oxidize fats relative to intake (RER:FQ ratio) correlated with changes in MetFlex. Moreover, improved fasting FATox correlated borderline significantly (*p* = 0.08) with increased PUFA intake, but not SFA. The secondary analyses, however, indicate improvements in MetFlex following sedentary time reduction without changes in dietary outcomes or differences in fat intake compared to continued high sedentary time (data not shown), suggesting that diet was not the driving factor of MetFlex. Still, the results add to the existing evidence, which suggests potentially differential effects of specific types of fatty acids on fat utilization, further highlighting the importance of lipid metabolism in MetFlex.

Altogether, our interventional and cross‐sectional findings, together with previous exercise and bed rest studies, support the hypothesis of physical (in)activity as an important regulator of MetFlex. The effects are likely related to lipid metabolism, mitochondrial function, and insulin sensitivity, even independent of weight loss and energy balance [10, 46]. The beneficial effects of successfully reduced sedentary time on MetFlex and low‐intensity exercise FATox are relevant from a clinical perspective as well. Improving the ability to utilize available substrates for energy production prevents lipids and/or glucose from accumulating ectopically in the liver and muscles, for example, and subsequently impairing insulin signaling. More optimized substrate utilization thus aids in the regulation of body weight and composition, as well as insulin sensitivity, as also reflected by the relationship between improvements in MetFlex and insulin sensitivity during the intervention. The findings are therefore clinically important in terms of the prevention of chronic metabolic diseases such as type 2 diabetes in individuals at high risk.

Future studies should aim to determine the mechanisms behind inactivity‐induced metabolic inflexibility and the effects of PA on MetFlex responses to different physiological challenges. Studies should also continue to explore the potential of increased standing and LPA as feasible, low‐barrier health‐enhancing strategies in addition to the established role of MVPA, and future intervention studies targeting sedentary behavior reduction in risk populations could consider adding a (light‐intensity) PA component. Findings from studies involving particularly inactive risk populations could aid in the development of intervention targets for the prevention of chronic diseases that could then be implemented in clinical settings and have important public health implications.

### 4.1 Strengths and Limitations

The key strength is the combination of rigorous methodology including a 6‐month, free‐living, randomized controlled design with continuous accelerometry, and MetFlex assessment with both HEC and (a more physiological) exercise challenge. The unintended behavioral changes in CON, possibly due to a participation effect and an increased awareness of health behaviors, may have affected the results and can be considered limitations. Although not the intention, the mobile app and the ability to see the accumulated time (and changes) in different behaviors may have tempted CON participants to modify their behaviors. The use of the app in CON as well was necessary, however, since the accelerometer data was transferred and stored to the cloud server for analysis only if the app was regularly accessed. The nature of the study—a behavioral health intervention—may also have led to recruitment bias, since volunteers for such a study may represent a more motivated group of the target population, and thus not be the most representative sample. The specific population of sedentary and inactive, middle‐aged Finnish adults with metabolic syndrome can limit the generalizability of the findings as well. However, given the prevalence of sedentary lifestyles and overweight and obesity, the findings may also be applicable on a wider scale, including younger populations (below the target age 40–65 years in this study) in which metabolic syndrome is increasingly prevalent. The timing of the second calorimetry measurement may be considered another limitation, as it was started relatively shortly after the initiation of HEC. However, during the 30‐ to 60‐min period of HEC when the second calorimetry measurement was performed, the mean blood glucose concentration was 5.0 mmol/L with a CV of 5.5% (SE 0.6), which was considered indicative of steady‐state conditions. Moreover, as our secondary analyses and cross‐sectional findings, but not primary intervention analyses, suggest a link between reduced sedentary time and improved MetFlex, more research is needed to confirm the causality of the relationship.

## 5 Perspective

This 6‐month free‐living sedentary time reduction intervention suggests benefits to metabolic flexibility and substrate oxidation with a successful reduction of sedentary time and increased standing. Modifying daily activity behaviors, without exercise training per se, may positively affect energy metabolism and help prevent a further deterioration of metabolic outcomes in populations with an increased cardiometabolic risk. However, more substantial improvements in overall metabolic health are likely achieved through an increased PA volume and intensity. The findings deepen the understanding of the determinants of energy metabolism regulation and highlight the role of daily sitting and habitual PA behaviors in the maintenance of metabolic health.

## Author Contributions

T.S., K.K.K., T.V., J.K., and I.H. conceived and designed the research. T.G., T.S., S.L., M.K., P.K., M.S., N.H., and K.L. performed experiments. T.G., T.S., S.L., H.V.‐Y., J.N., E.L., H.S., and I.H. analyzed data and interpreted the results of experiments. T.G. prepared figures and drafted the manuscript. All authors edited and revised the manuscript and approved the final version of the manuscript.

## Ethics Statement

The protocol was approved by the Ethics Committee of the Hospital District of Southwest Finland (16/1801/2017).

## Consent

Informed consent was obtained from all participants in writing.

## Conflicts of Interest

Outside of this work, J.K. has received consultancy fees from GE Healthcare and AstraZeneca and speaker fees from GE Healthcare, Bayer, Lundbeck, Boehringer‐Ingelheim, and Merck; T.S. has received a speaker fee from Pihlajalinna. The other authors declare that they have nothing to disclose.

## Supporting information


**Appendix S1:** sms70113‐sup‐0001‐AppendixS1.pdf.

## Data Availability

The data that support the findings of this study are available from the corresponding author upon reasonable request.
