# Supplementary material for: Successfully Reducing Sitting Time Can Improve Metabolic Flexibility
Source: Scand J Med Sci Sports. 2025 Aug 9;35(8):e70113. doi: 10.1111/sms.70113 (PMC12334929; doi:10.1111/sms.70113)
Supplement: Supplementary file 1 — Appendix S1: sms70113‐sup‐0001‐AppendixS1.pdf. [file SMS-35-e70113-s001.pdf]

Successfully reducing sitting time can improve metabolic flexibility

Taru Garthwaite[1], Tanja Sjöros[1], Saara Laine[1], Mikko Koivumäki[1], Henri Vähä-Ypyä[2], Jooa Norha[1], Petri Kallio[3,4], Maria Saarenhovi[3], Eliisa Löyttyniemi[5], Harri Sievänen[2], Noora Houttu[6], Kirsi Laitinen[6], Kari K. Kalliokoski[1], Tommi Vasankari[2,7], Juhani Knuuti[1], Ilkka Heinonen[1]

[1] Turku PET Centre, University of Turku, Åbo Akademi University, and Turku University Hospital, Turku, Finland
[2] The UKK Institute for Health Promotion Research, Tampere, Finland
[3] Department of Clinical Physiology and Nuclear Medicine, University of Turku and Turku University Hospital, Turku, Finland
[4] Paavo Nurmi Centre and Unit for Health and Physical Activity, University of Turku, Turku, Finland
[5] Department of Biostatistics, University of Turku and Turku University Hospital, Turku, Finland
[6] Institute of Biomedicine and Food and Nutrition Research Center, University of Turku, Turku, Finland
[7] Faculty of Medicine and Health Technology, Tampere University, Tampere, Finland

Supplemental File 1. Supplemental Figures 1 and 2 and Supplemental Tables 1-4.

Supplemental Figure 1. CONSORT Flow Diagram.

Supplemental Figure 2. The change in mean daily sedentary time (h/day) from baseline to 6 months of each participant with valid accelerometer data during the intervention (n=56). Green bars represent the participants in the intervention group, and blue bars represent the control group. The interindividual variation in mean daily sedentary time changes during the intervention was examined using proportions (%) of accelerometer wear time/day but is presented in h/day for easier interpretation.

Modified from Sjöros, T. The Effects of Reducing Sedentary Behaviour on Wholebody and Skeletal Muscle Insulin Sensitivity. Dissertation. Turku, Finland: University of Turku; 2022. https://urn.fi/URN:ISBN:978-951-29-8869-3

Supplemental Table 1. Intervention effects on insulin-stimulated metabolic flexibility and substrate oxidation within and between groups from baseline to 6 months.

Supplemental Table 2. Intervention effects on metabolic flexibility and substrate oxidation during exercise within and between groups from baseline to 6 months.

Supplemental Table 3. Intervention effects on metabolic flexibility during insulin-stimulation and exercise within and between participants who reduced sedentary time ≥ 30 min/day vs. continuously sedentary participants from baseline to 6 months.

Supplemental Table 4. Correlations (Spearman's rho) between changes (Δ) in metabolic flexibility, metabolic and anthropometric outcomes, and physical activity during the intervention.

Supplemental Figure 1. CONSORT Flow Diagram.

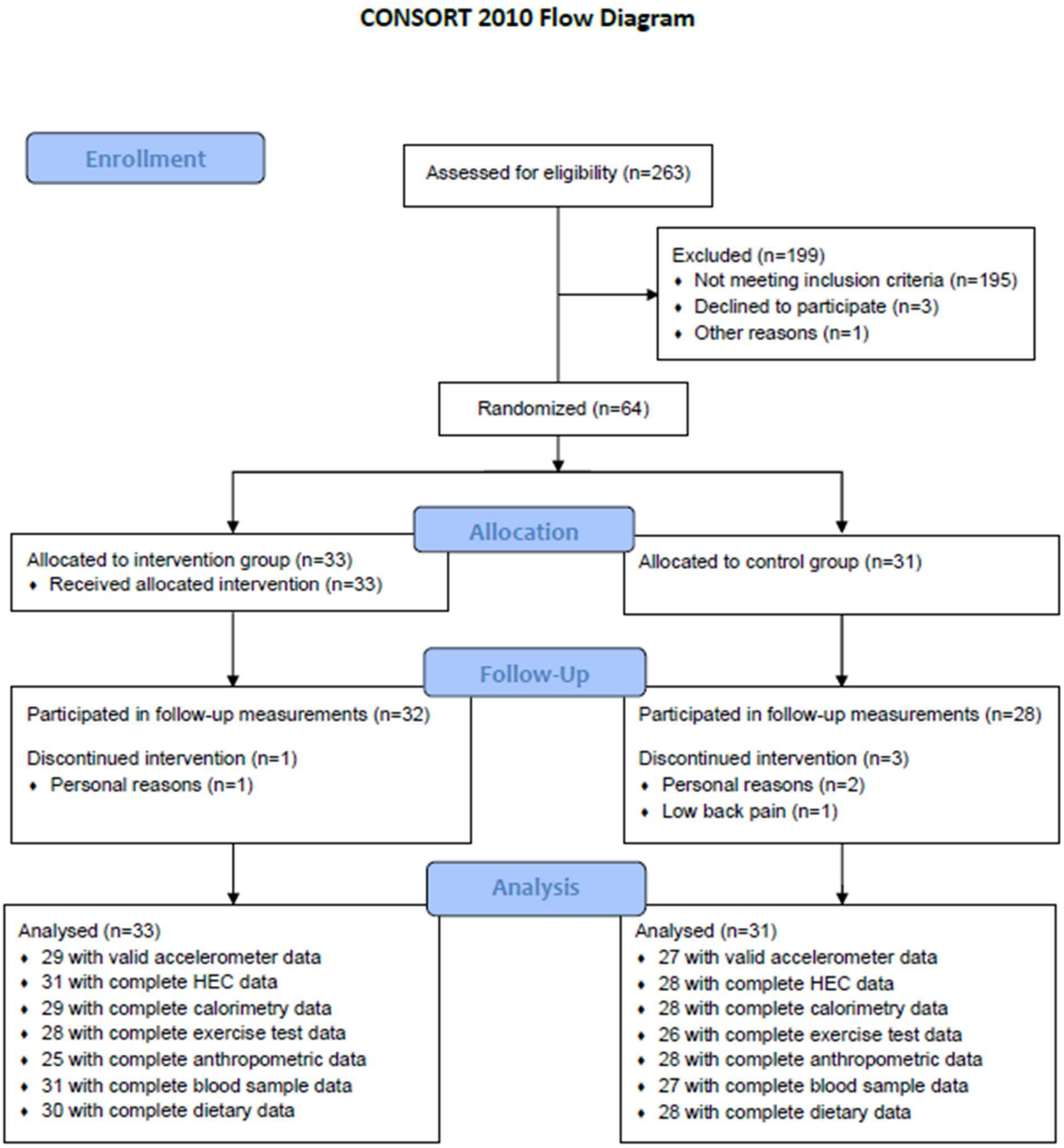

Supplemental Figure 2. The change in mean daily sedentary time (h/day) from baseline to 6 months of each participant with valid accelerometer data during the intervention (n=56). Green bars represent the participants in the intervention group, and blue bars represent the control group. The interindividual variation in mean daily sedentary time changes during the intervention was examined using proportions (%) of accelerometer wear time/day but is presented in h/day for easier interpretation.

Modified from Sjöros, T. The Effects of Reducing Sedentary Behaviour on Wholebody and Skeletal Muscle Insulin Sensitivity. Dissertation. Turku, Finland: University of Turku; 2022. https://urn.fi/URN:ISBN:978-951-29-8869-3

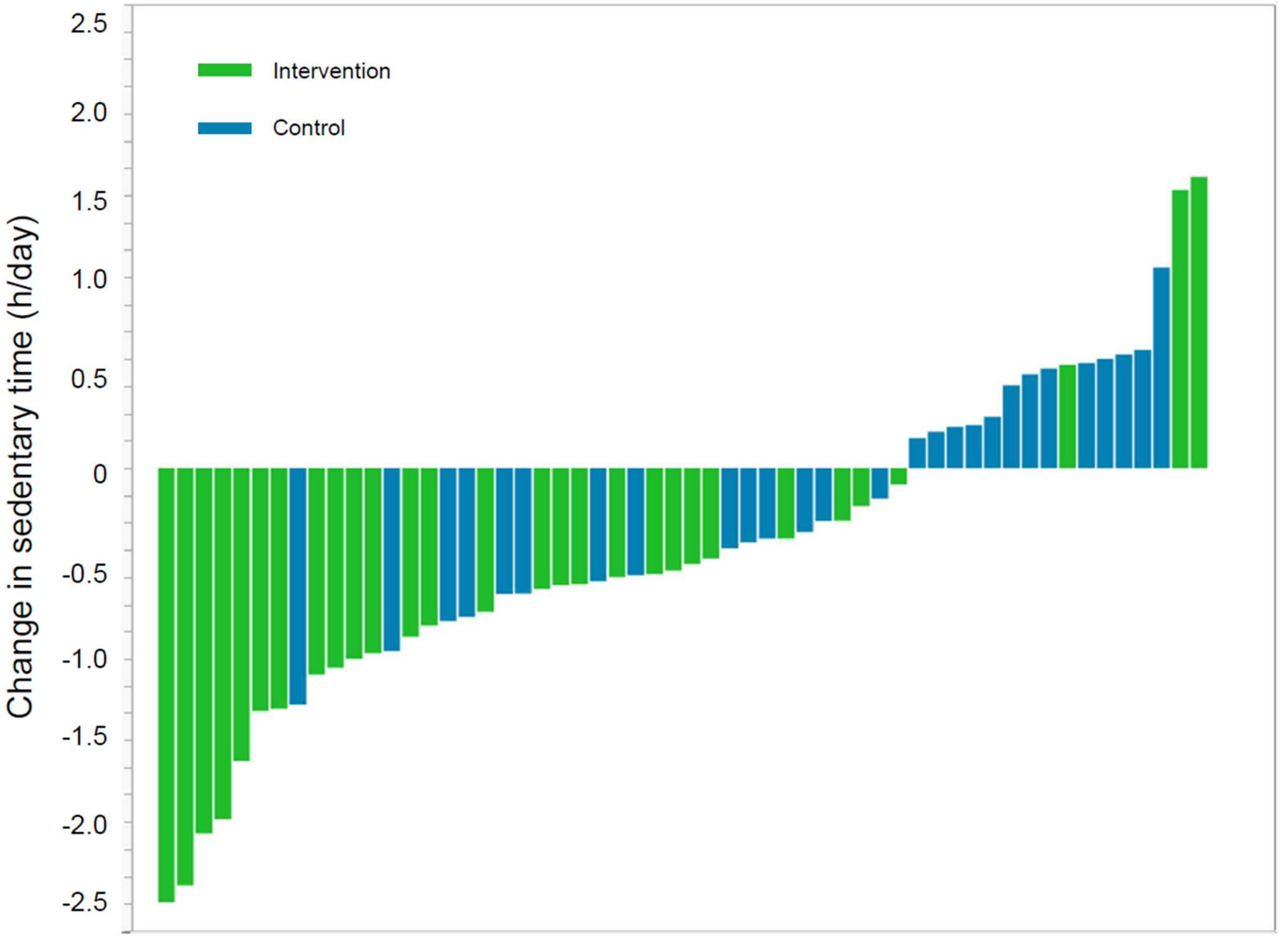

Supplemental Table 1. Intervention effects on insulin-stimulated metabolic flexibility and substrate oxidation within and between groups from baseline to 6 months. RER = respiratory exchange ratio; HEC = hyperinsulinemic euglycemic clamp; CHO = carbohydrate.

		**Intervention (n=33)**		**Control (n=31)**		**Difference between groups (95 % CI) from baseline to 6 months**	**p-values [a]**		
		Baseline [b]	6 months [b]	Baseline [b]	6 months [b]	Control−Intervention [b]	Group	Time	Group x Time
RER	Fasting	0.94 (0.91, 0.97)	0.90 (0.87, 0.93)	0.90 (0.87, 0.93)	0.91 (0.88, 0.95)	−0.05 (−0.12, 0.00)	0.45	0.32	0.11
	HEC	0.93 (0.90, 0.96)	0.91 (0.87, 0.94)	0.90 (0.86, 0.93)	0.90 (0.86, 0.93)	−0.03 (−0.09, 0.03)	0.25	0.48	0.38
ΔRER (HEC−fasting) [c]		−0.02 (−0.04, 0.01)	0.01 (−0.02, 0.03)	−0.01 (−0.03, 0.02)	−0.01 (−0.04, 0.01)	0.03 (−0.01, 0.08)	0.75	0.50	0.18
CHOox (mg/kg/min)	Fasting	2.8 (2.4, 3.1)	2.3 (2.0, 2.6)	2.2 (1.9, 2.5)	2.2 (1.9, 2.6)	-0.5 (-1.1, 0.1)	0.10	0.11	0.10
	HEC	2.8 (2.4, 3.2)	2.5 (2.1, 2.9)	2.3 (1.9, 2.7)	2.2 (1.8, 2.6)	-0.2 (-0.9, 0.4)	0.11	0.20	0.48
FATox (mg/kg/min)	Fasting	0.3 (0.1, 0.4)	0.4 (0.3, 0.6)	0.5 (0.3, 0.6)	0.4 (0.3, 0.6)	0.2 (-0.1, 0.4)	0.25	0.34	0.12
	HEC	0.3 (0.2, 0.5)	0.4 (0.3, 0.6)	0.5 (0.3, 0.6)	0.5 (0.3, 0.6)	0.1 (-0.2, 0.4)	0.28	0.59	0.53

[a] = Group: the main effect of group differences; Time: the main effect of time; Group x Time: the interaction between the two main effects.
[b] = model-based means (95 % CI).

Supplemental Table 2. Intervention effects on metabolic flexibility and substrate oxidation during exercise within and between groups from baseline to 6 months.

		**Intervention (n=33)**		**Control (n=31)**		**Difference between groups (95 % CI) from baseline to 6 months**	**p-values [a]**		
		Baseline [b]	6 months [b]	Baseline [b]	6 months [b]	Control−Intervention [b]	Group	Time	Group x Time
RER	Low-intensity	0.73 (0.72, 0.75)	0.73 (0.71, 0.74)	0.74 (0.73, 0.76)	0.72 (0.70, 0.74)	0.02 (-0.01, 0.04)	0.69	0.05	0.21
	Maximal	1.12 (1.09, 1.14)	1.12 (1.10, 1.15)	1.12 (1.09, 1.14)	1.11 (1.09, 1.14)	0.01 (-0.02, 0.04)	0.75	0.96	0.49
ΔRER (maximal – low-intensity)		0.39 (0.36, 0.41)	0.40 (0.37, 0.42)	0.37 (0.35, 0.40)	0.39 (0.36, 0.42)	-0.01 (-0.04, 0.03)	0.57	0.13	0.64
CHOox (mg/kg/min)	Low-intensity	2.2 (1.7, 2.7)	2.1 (1.6, 2.7)	2.2 (1.7, 2.7)	2.2 (1.6, 2.7)	-0.1 (-1.1, 1.0)	0.95	0.77	0.88
	Maximal	38.4 (34.5, 42.4)	41.1 (37.2, 45.0)	39.0 (35.1, 42.9)	37.6 (33.6, 41.6)	4.1 (0.3, 7.8)	0.58	0.51	**0.03**
FATox (mg/kg/min)	Low-intensity	2.3 (1.9, 2.6)	2.3 (1.9, 2.6)	2.5 (2.1, 2.8)	2.3 (1.9, 2.6)	0.2 (-0.4, 0.8)	0.54	0.55	0.59
	Maximal	-4.0 (-5.1, -3.0)	-4.6 (-5.6, -3.6)	-4.1 (-5.1, -3.1)	-3.9 (-4.9, -2.8)	-0.8 (-2.2, 0.6)	0.61	0.66	0.23

RER = respiratory exchange ratio; CHO = carbohydrate.

[a] = Group: the main effect of group differences; Time: the main effect of time; Group x Time: the interaction between the two main effects.

[b] = model-based means (95 % CI).

Supplemental Table 3. Intervention effects on metabolic flexibility during insulin-stimulation and exercise within and between participants who reduced sedentary time ≥ 30 min/day vs. continuously sedentary participants from baseline to 6 months.

		Reduced sedentary time (n=34)		Continuously sedentary (n=30)		Difference between groups (95 % CI) from baseline to 6 months	p-values [a]		
		Baseline [b]	6 months [b]	Baseline [b]	6 months [b]	Continuously sedentary − Reducers [b]	Group	Time	Group x Time
RER	Fasting	0.95 (0.92, 0.98)	0.90 (0.87, 0.94)	0.89 * (0.85, 0.92)	0.91 (0.87, 0.94)	−0.06 (−0.12, 0.00)	0.07	0.37	**0.04**
	HEC	0.93 (0.89, 0.96)	0.91 (0.88, 0.95)	0.90 (0.87, 0.94)	0.89 (0.86, 0.93)	−0.01 (−0.07, 0.05)	0.25	0.48	0.81
ΔRER (HEC−fasting) [c]		−0.02 (−0.05, 0.00)	0.01 (−0.02, 0.03)	0.00 (−0.02, 0.03)	−0.01 (−0.04, 0.01)	0.04 (0.00, 0.09)	0.84	0.54	**0.04**
RER	Low-intensity	0.73 (0.72, 0.75)	0.73 (0.71, 0.74)	0.74 (0.72, 0.76)	0.72 (0.70, 0.73)	0.02 (−0.01, 0.04)	0.87	**0.04**	0.19
	Maximal	1.12 (1.09, 1.14)	1.12 (1.10, 1.14)	1.12 (1.09, 1.14)	1.12 (1.09, 1.14)	0.01 (−0.03, 0.04)	0.97	0.95	0.72
ΔRER (maximal −low-intensity)		0.38 (0.36, 0.41)	0.39 (0.37, 0.42)	0.37 (0.35, 0.40)	0.40 (0.37, 0.42)	−0.01 (−0.05, 0.02)	0.92	0.11	0.41

RER = respiratory exchange ratio; HEC = hyperinsulinemic euglycemic clamp.

[a] = Group: the main effect of group differences; Time: the main effect of time; Group x Time: the interaction between the two main effects. Bold p-values indicate statistical significance ($p < 0.05$)
[b] = model-based means (95 % CI).
* = Tukey's $p < 0.05$ between groups at baseline.

Supplemental Table 4. Spearman's correlations between changes (Δ) in metabolic flexibility, metabolic and anthropometric outcomes, and physical activity during the intervention.

	Δ Fasting RER	Δ HEC RER	Δ HEC MetFlex	Δ Low-intensity exercise RER	Δ Maximal exercise RER	Δ Exercise MetFlex	Δ Weight, kg	Δ BMI, kg/m2	Δ Waist circumference, cm	Δ Body fat-%	Δ Fat mass, kg	Δ Fat-free mass, kg	Δ Fasting insulin, pmol/L	Δ Fasting glucose, mmol/L	Δ Whole-body glucose uptake, mg/kg/min	Δ Fasting free fatty acids, mmol/L	Δ Fasting lactate, mmol/L	Δ HEC free fatty acid suppression, %	Δ HEC lactate increase, %	Δ Fasting triglycerides, mmol/L	Δ Sedentary time, %/wear time	Δ Standing, %/wear time	Δ LPA, %/wear time	Δ MVPA, %/wear time	Δ Steps/day
Δ Fasting RER	--																								
ΔHEC RER	**0.77** **	--																							
Δ HEC MetFlex	**-0.33** *	0.26	--																						
Δ Low-intensity exercise RER	0.12	0.09	-0.10	--																					
Δ Maximal exercise RER	**0.29** *	**0.41** **	0.14	**0.33** *	--																				
Δ Exercise MetFlex	0.08	0.27	0.21	**-0.47** **	**0.60** **	--																			
Δ Weight, kg	-0.10	-0.23	-0.08	-0.06	**-0.37** **	**-0.34** *	--																		
Δ BMI, kg/m2	-0.12	-0.24	-0.07	-0.06	**-0.37** **	**-0.34** *	**1.00** **	--																	
Δ Waist circumference, cm	0.05	-0.01	-0.10	-0.09	-0.14	-0.11	**0.47** **	**0.47** **	--																
Δ Body fat-%	-0.07	-0.17	-0.09	0.15	-0.20	**-0.34** *	**0.28** *	**0.29** *	0.16	--															

Δ Fat mass, kg	-0.09	-0.21	-0.09	0.03	**-0.35** *	**-0.36** **	**0.65** **	**0.66** **	**0.33** *	**0.88** **	--														
Δ Fat-free mass, kg	-0.03	-0.02	0.03	-0.19	-0.11	0.04	**0.43** **	**0.42** **	0.15	**-0.68** **	**-0.32** *	--													
Δ Fasting insulin, pmol/L	-0.13	-0.05	0.05	-0.23	-0.06	0.10	**0.31** *	**0.29** *	0.24	-0.04	0.10	0.22	--												
Δ Fasting glucose, mmol/L	-0.01	0.02	0.11	-0.16	-0.09	0.00	0.14	0.15	0.10	-0.03	0.00	0.19	**0.28** *	--											
Δ Whole-body glucose uptake, mg/kg/min	0.11	**0.32** *	**0.27** *	-0.03	0.16	0.16	**-0.53** **	**-0.53** **	**-0.33** *	-0.09	**-0.27** *	**-0.27** *	**-0.29** *	**-0.26** *	--										
Δ Fasting free fatty acids, mmol/L	-0.19	**-0.29** *	-0.14	-0.12	-0.26	-0.11	**0.28** *	**0.29** *	0.18	0.02	0.15	0.14	-0.08	-0.20	**-0.27** *	--									
Δ Fasting lactate, mmol/L	**0.37** **	0.13	**-0.28** *	-0.19	0.09	0.21	**0.27** *	**0.27** *	0.07	-0.00	0.13	**0.26** *	0.17	0.18	-0.16	-0.13	--								
Δ HEC free fatty acid suppression, %	0.03	-0.03	-0.12	0.10	-0.07	-0.25	-0.12	-0.13	-0.13	-0.03	-0.06	-0.03	-0.10	-0.03	**0.30** *	0.23	-0.18	--							
Δ HEC lactate increase, %	-0.19	-0.06	0.24	0.26	-0.13	**-0.30** *	-0.22	-0.23	-0.11	-0.06	-0.17	-0.13	-0.24	-0.03	**0.34** **	0.06	**-0.67** **	0.18	--						
Δ Fasting triglycerides, mmol/L	0.16	-0.05	-0.22	-0.15	0.05	0.13	**0.26** *	0.26	0.24	-0.09	0.02	0.25	0.15	0.01	-0.24	0.15	0.21	-0.14	-0.05	--					
Δ Sedentary time, %/wear time	0.15	-0.02	-0.24	-0.15	-0.21	-0.04	**0.36** **	**0.33** *	0.20	0.13	0.26	0.08	0.22	-0.09	**-0.38** **	-0.03	0.14	-0.17	-0.12	0.07	--				
Δ Standing, %/wear time	-0.18	0.07	**0.30** *	0.20	0.23	0.03	-0.24	-0.22	0.04	-0.18	-0.24	0.04	-0.20	0.20	**0.32** *	-0.04	-0.17	0.13	0.21	-0.04	**-0.76** **	--			
Δ LPA, %/wear time	0.00	0.03	0.19	-0.08	0.06	0.06	**-0.32** *	**-0.30** *	-0.23	0.08	-0.09	**-0.32** *	-0.20	-0.09	**0.31** *	0.08	-0.17	0.17	0.04	-0.09	**-0.65** **	0.20	--		

Δ MVPA, %/wear time	-0.10	-0.13	0.04	0.22	0.12	-0.12	-0.25	-0.25	-0.26	-0.09	-0.17	-0.11	-0.07	-0.05	0.20	0.20	-0.18	0.23	0.08	-0.16	-0.66**	0.25	0.52**	--	
Δ Steps/day	0.02	0.00	0.00	0.15	0.14	-0.05	-0.36**	-0.36**	-0.28	-0.29*	-0.37**	0.00	-0.22	-0.15	0.30*	0.11	-0.15	0.27	0.14	-0.26	-0.60**	0.29*	0.43**	0.73**	--

RER = respiratory exchange ratio; HEC = hyperinsulinemic euglycemic clamp; MetFlex = metabolic flexibility; LPA = light-intensity physical activity; MVPA = moderate-to-vigorous physical activity.

Bold p-values indicate statistical significance; * = p < 0.05, ** = p < 0.01.
